# Neurotrophic Receptor Tyrosine Kinase 3 as a Prognostic Biomarker in Breast Cancer Using Bioinformatic Analysis

**DOI:** 10.3390/medicina61030474

**Published:** 2025-03-08

**Authors:** Jeongmin Choi, Jongwan Kim, Yong Wook Jung, Jong Ho Park, Jae-Ho Lee

**Affiliations:** 1 Medical Course, School of Medicine, Keimyung University, 1095 Dalgubeol-daero, Daegu 42601, Republic of Korea; jmchoi0525@naver.com; 2 Department of Anatomy, College of Medicine, Dongguk University, Gyeongju 38066, Republic of Korea; 3 Department of Anatomy, School of Medicine, Keimyung University, 1095 Dalgubeol-daero, Daegu 42601, Republic of Korea; jpark@dsmc.or.kr

**Keywords:** breast cancer, neurotrophic receptor tyrosine kinase 3, prognosis, immune cells, biomarker

## Abstract

*Background and Objectives*: Neurotrophic receptor tyrosine kinase 3 (*NTRK3*) is a member of the tropomyosin receptor kinase family of receptor tyrosine kinases, which play a crucial role in neural development. However, owing to the limited number of studies about *NTRK3* and cancer, we aimed to investigate *NTRK3* as a potential prognostic marker for breast cancer (BC). *Materials and Methods*: We conducted a comprehensive analysis of *NTRK3* expression in BC using the Tumor Immune Estimation Resource, Gene Expression Profiling Interactive Analysis 2, and Kaplan–Meier Plotter databases. We also explored the association between *NTRK3* expression and tumor-infiltrating immune cells. *Results*: Low *NTRK3* expression showed poorer prognosis in BC, as well as with T stage, pathology, and the Luminal subtype. In BC (BRCA), *NTRK3* was positively correlated with CD4+ T cell, CD8+ T cell, macrophage, and neutrophil infiltration. *Conclusions*: These results suggest that *NTRK3* may serve as a prognostic biomarker and provide novel insights into tumor immunology in BC. Therefore, *NTRK3* represents a potential diagnostic and therapeutic target for BC treatment.

## 1. Introduction

Breast cancer (BC) represents the most common malignant tumor in women and is the leading cause of cancer-related deaths [1]. However, most people do not exhibit symptoms when the cancer is still at an early stage. BC commonly presents as a painless lump in the breast. Early detection and timely treatment improve effectiveness and tolerability [2]. Moreover, identifying prognostic biomarkers is crucial for better BC treatment outcomes. Additionally, understanding the molecular mechanisms underlying BC progression can lead to the development of novel biomarkers that may offer better prognostic accuracy.

Neurotrophic receptor tyrosine kinase (NTRK) genes (*NTRK1*–*NTRK3*) as proto-oncogenes, are found in various tumors [3]. Tropomyosin receptor kinases (TRK), also known as neurotrophic tyrosine kinase receptors, play essential roles in neuronal biology by mediating neurotrophin-activated signaling. The TRK receptor family comprises three transmembrane receptors: TrkA, TrkB, and TrkC, which are encoded by *NTRK1*, *NTRK2*, and *NTRK3*, respectively [4]. Evidence suggests that TrkC plays a role in cancer. In breast, pancreatic, and prostate cancers, the expressions of TrkC subtype were associated with their carcinogenesis [5].

*NTRK3* is a gene that encodes a receptor tyrosine kinase of the TRK family, which plays an important role in neural development by mediating the effects of neurotrophins [6]. In cancer, *NTRK3* is often involved in gene fusions, where part of the *NTRK3* gene is joined to another gene, leading to the production of a fusion protein that can drive tumorigenesis. These fusions result in the overactivation of the TRK signaling pathway, promoting cancer progression. Despite its role, research on the correlation between *NTRK3* and cancer remains limited, necessitating further investigation [7].

The immune system plays a critical role in tumor onset and progression. *NTRK3* is expressed by cells of the lymphatic system, such as T lymphocytes and neutrophils, as well as by myeloid immune cells, such as macrophages. Additionally, *NTRK3* regulates the transcriptional networks for cell maintenance and differentiation [8]. However, the relation between *NTRK3* and immune cell infiltration in BC remains unknown. Therefore, we studied a correlation between *NTRK3* and tumor-infiltrating immune cells in various cancers, especially BC.

In this article, we evaluated the prognostic value of *NTRK3* by analyzing its association with clinicopathological features and tumor-infiltrating immune cells (TIICs) in BC. *NTRK3* expression and its prognostic significance was examined using publicly available databases, including the Tumor Immune Estimation Resource (TIMER; https://cistrome.shinyapps.io/timer/, accessed on 1 September 2024), OncoLnc (OSLIHC; http://bioinfo.henu.edu.cn/DatabaseList., accessed on 16 September 2024), and UALCAN (http://ualcan.path.uab.edu, accessed on 8 September 2024). Our results indicate that *NTRK3* may serve as a viable therapeutic target and a promising candidate for immunotherapy in patients with BC.

## 2. Materials and Methods

### 2.1. TIMER Database Analysis

TIMER is used to analyze tumor-infiltrating immune cells in BC [9]. TIMER assesses tumor-infiltrating immune cells based on statistical analyses of gene expression profiles [10]. The correlation between *NTRK3* and infiltrating immune cells, such as macrophages, neutrophils, CD4+ T cells, and CD8+ T cells, was analyzed in BC. We investigated the relationship between NTRK3 expression and TIICs, along with the prognostic implications of this association.

### 2.2. OSLIHC Database Analysis

The OSLIHC database provides a platform for researchers to identify new prognostic biomarkers and enables the development of novel targeted therapies for various cancers. Various survival outcomes, such as overall survival (OS), disease-free interval, progression-free interval (PFI), and disease-specific survival (DSS), were obtained in OSLIHC.

### 2.3. UALCAN Database Analysis

The UALCAN database is a web-based tool for cancer data analysis that utilizes The Cancer Genome Atlas (TCGA) Level 3 RNA-Seq data and clinical data. This tool enables users to analyze the relative expression of specific genes across both tumor and normal samples as well as across different tumor subgroups. These subgroups can be classified based on various clinicopathological features, such as sex, age, race, histological subtype, tumor stage, grade, nodal metastasis, and *TP53* mutation status.

### 2.4. Statistical Analysis

Data were statistically analyzed by SPSS software (version 25.0; IBM SPSS, Armonk, NY, USA). Clinicopathological characteristics, including age, sex, and pathological TNM stage, were analyzed using the chi-square test. The TNM stage was determined according to the seventh edition of the American Joint Committee on Cancer staging system. Survival results were presented as hazard ratios (HRs) by the log-rank test. A *p*-value of less than 0.05 from the log-rank test indicated a significant difference in survival times. The TIMER database was used to assess the correlation between gene expression levels and immune signature scores using Spearman’s correlation coefficients. All reported results included *p*-values, with values below 0.05 deemed to indicate statistical significance.

## 3. Results

### 3.1. Assessment of NTRK3 Expression in Different Cancer and Normal Tissues

**Figure 1:**
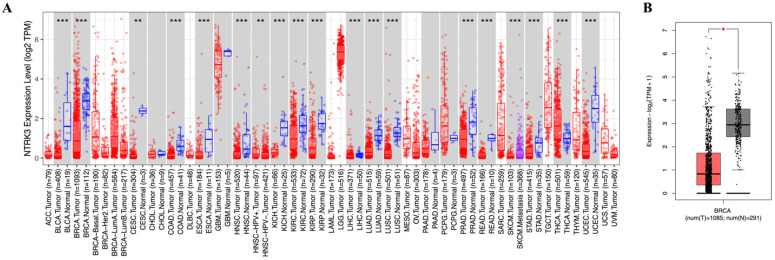
mRNA expression levels of NTRK3 in various types of cancer, including BC. (**A**) High or low expression of NTRK3 in tumor tissues compared with normal tissues analyzed using the TIMER database. (**B**) Low expression of NTRK3 in tumor tissues compared with normal tissues analyzed using the GEPIA2 database. * *p* < 0.05, ** *p* < 0.01 and *** *p* < 0.001.

TIMER data from TCGA showed *NTRK3* expression level in specific tumor types. Significant differences in *NTRK3* expression between normal and tumor tissues were observed in bladder carcinoma (*p* < 0.001), breast carcinoma (*p* < 0.001), cervical squamous cell carcinoma (*p* < 0.01), colon adenocarcinoma (*p* < 0.001), esophageal cancer (*p* < 0.001), head and neck squamous cell carcinoma (*p* < 0.001), kidney chromophobe carcinoma (*p* < 0.001), kidney renal papillary cell carcinoma (*p* < 0.001), liver hepatocellular carcinoma (*p* < 0.001), lung adenocarcinoma (*p* < 0.001), lung squamous cell carcinoma (*p* < 0.001), prostate adenocarcinoma (*p* < 0.001), rectum adenocarcinoma (*p* < 0.001), skin cutaneous melanoma (*p* < 0.001), stomach adenocarcinoma (*p* < 0.001), thyroid carcinoma (*p* < 0.001), and uterine corpus endometrial carcinoma (*p* < 0.001) (Figure 1A). In addition, the GEPIA2 database revealed that *NTRK3* expression was downregulated in BC (Figure 1B).

**Figure 2:**
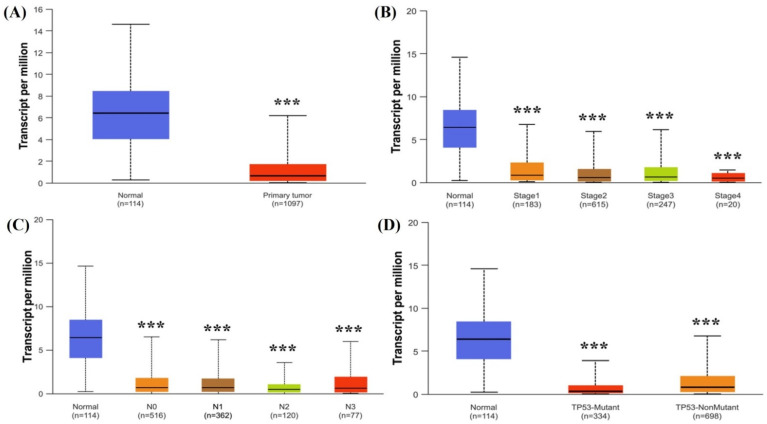
mRNA expression levels of NTRK3 expression in BC according to clinicopathologic characteristics: (**A**) tumor and normal; (**B**) stage; (**C**) N stage; (**D**) TP53 status. *** *p* < 0.001.

The UALCAN database showed clinical characteristics of *NTRK3* expression in BC to validate the TIMER results. *NTRK3* expression was associated with tumor size, tumor stage, lymph node status, and TP53 mutation in BC (Figure 2).

### 3.2. Clinicopathological Characteristics of NTRK3 in BC

Patients were divided into two subgroups according to the median values of *NTRK3* expression to evaluate its clinical significance in BC. The clinical value of *NTRK3* is summarized in Table 1. Lower *NTRK3* expression was associated with higher T stage (*p* = 0.002), Black and Asian racial groups (*p* = 0.011), invasive ductal carcinoma (*p* < 0.001), and luminal subtype (*p* < 0.001) significantly. Although not statistically significant, *NTRK3* expression was related to N stage (*p* = 0.09). No other clinical characteristics were associated with *NTRK3* expression in BC patients.

### 3.3. Prognostic Value of NTRK3 in BC

**Figure 3:**
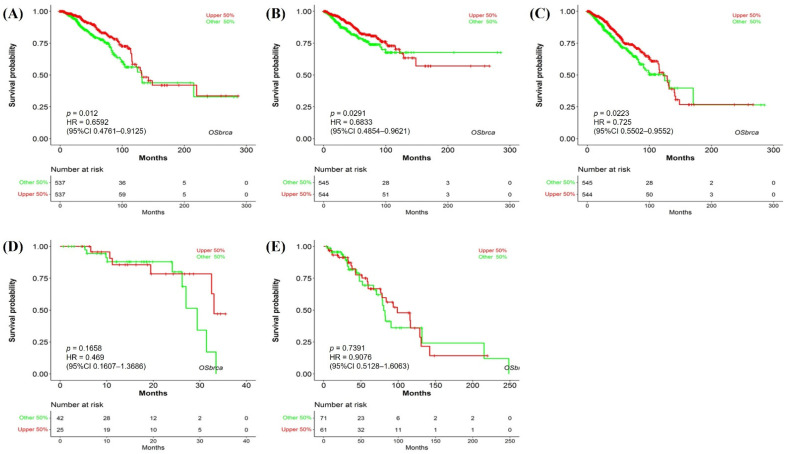
Prognostic significance of NTRK3 in BRCA. (**A**) Overall survival; (**B**) progression-free interval; (**C**) progression-free survival; (**D**) disease-free interval; (**E**) disease-specific survival.

We evaluated the correlation between *NTRK3* expression and BC prognosis using the OSLIHC database. Survival rates, including OS, disease-free interval, PFI, progression-free survival, and DSS, were analyzed based on *NTRK3* expression in BC. As result, *NTRK3* expression was significantly associated with a better prognosis in BC, as evidenced by OS (HR = 0.6592, *p* = 0.012, Figure 3A), PFI (HR = 0.6833, *p* = 0.0291, Figure 3B), and progression-free survival (HR = 0.725, *p* = 0.0223, Figure 3C). The disease-free interval and DSS did not have any difference according to *NTRK3* expression. Our results demonstrate that the downregulation of *NTRK3* expression predicts a poor prognosis in BC.

### 3.4. Association of NTRK3 Expression with Immune Cell Infiltration in BC

**Figure 4:**
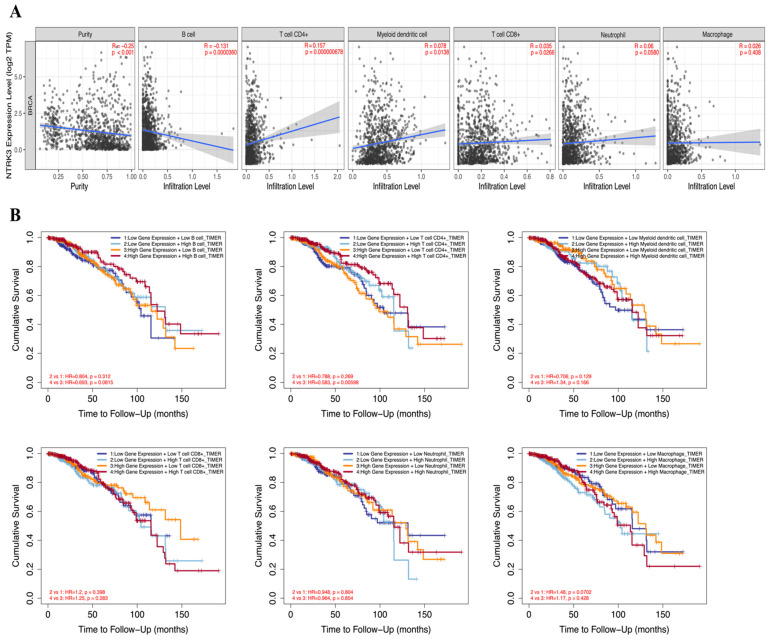
Correlation between NTRK3 expression and infiltrating immune cells in BRCA. (**A**) Scatterplots showing the correlation between NTRK3 and TIICs, including B cell, CD4+ T cells, dendritic cells, CD8+ T cells, neutrophils, and macrophages were analyzed using the TIMER database. (**B**) The prognostic value NTRK3 and TIICs was analyzed using the TIMER database.

Immune cell infiltration accelerates cancer progression and affects survival. We examined the correlation between *NTRK3* expression and TIICs in BC using the TIMER database (Figure 4A). *NTRK3* was positively correlated with dendritic cells (r = 0.078, *p* = 0.014), CD4+ T cells (r = 0.157, *p* < 0.001), and CD8+ T cells (r = 0.035, *p* = 0.027). Conversely, *NTRK3* was negatively correlated with B cell infiltration levels (r = −0.131, *p* < 0.001) in BC (Figure 4A). Subsequently, we examined the associations among *NTRK3* expression, prognosis, and TIIC infiltration in BC. High *NTRK3* expression with low CD4+ T cell infiltration showed a poorer prognosis than high NTRK3 expression with high CD4+ T cell infiltration in BC (Figure 4B). Cumulative survival curve analysis indicated that immune cell infiltration was associated with *NTRK3* in BC and affected prognosis. These data suggest that *NTRK3* is a predictive gene for immune cell invasion in BC.

## 4. Discussion

Genetic studies are crucial for identifying therapeutic targets in BC because they provide insights into the molecular mechanisms that drive tumor growth and progression [11]. By analyzing genetic characteristics, clinicians can identify specific mutations, gene amplifications, or fusions associated with cancer progression [12]. This enables the development of targeted therapies aimed at these genetic alterations, improving treatment outcomes and reducing side effects. Moreover, genetic analysis helps personalize treatment plans, as not all BCs are identical [13]. For example, HER2-positive BC can be treated with targeted therapies, such as trastuzumab, whereas hormone receptor-positive BC may benefit from hormone therapies. By understanding the genetic profile of each tumor, more precise and effective treatments can be developed, leading to improved prognosis and survival rates. Overall, genetic analysis is essential for identifying new therapeutic targets and advancing personalized medicine for BC treatment [14].

Recently, data from TCGA have played a pivotal role in advancing our understanding of the molecular landscape of this disease. TCGA provides a comprehensive multidimensional dataset that integrates genomic, transcriptomic, and clinical information from a large number of BC samples [15,16]. This valuable resource enables researchers to identify key genetic alterations, mutations, and expression patterns that drive tumorigenesis and contribute to therapeutic resistance. We investigated the clinical and prognostic value of *NTRK3* expression in BC using the TIMER and UALCAN databases in conjunction with TCGA data. Differential expression of *NTRK3* between tumors and normal tissues has been observed in various cancers, and *NTRK3* expression is lower in BC tissues than in normal adjacent tissues. A previous study showed that NTRK3 may be a potential tumor suppressor gene in cancer via inactivated epigenetic and genetic mechanisms [17]. Aberrant methylation patterns contribute to the downregulation of this tumor suppressor gene, silencing NTRK3 expression. However, Jin et al. [6] showed that its overexpression promoted breast tumor growth and metastasis. This discrepancy suggests that the role of NTRK3 in BC may be context-dependent, potentially varying based on tumor subtype, stage, or other molecular factors. Therefore, further investigation is needed to fully understand the dual roles of NTRK3 in BC and to identify the specific conditions under which its expression may either inhibit or enhance cancer progression.

*NTRK3* may be a critical factor in cancer progression, including gastric [18], thyroid [19], lung [20], glial [21], and BCs. These studies indicate that *NTRK3* might affect the AKT–mTOR signaling pathway, influencing cancer progression. However, our big data analysis showed that *NTRK3* expression was associated with better survival outcomes. Similar results have been reported in melanoma [22], neuroblastoma [23], and colorectal cancer [17]. These conflicting results indicate that the precise role of *NTRK3* in cancer progression remains unclear.

To elucidate the role of *NTRK3* in BC, we examined the clinicopathological characteristics of BC. Lower *NTRK3* expression was associated with a higher T stage, indicating larger tumor size and greater metastatic potential. Additionally, we found that lower *NTRK3* expression correlated with certain racial groups. Interestingly, this expression pattern was related to the luminal subtype. *NTRK3* expression was particularly low in patients with luminal B and HER2-enriched subtypes, which are associated with poorer prognosis [24,25]. These results may be attributed to the higher rates of local recurrence and reduced response to anti-hormonal treatment in these patients [24,25,26]. Therefore, new treatment options should be studied for luminal B and HER2-enriched subtypes based on *NTRK3* expression data.

The positive correlation between NTRK3 expression and dendritic cells, CD4+ T cells, and CD8+ T cells indicates that NTRK3 may be involved in modulating immune responses in the tumor microenvironment. Dendritic cells, CD4+ T cells, and CD8+ T cells play crucial roles in initiating and sustaining anti-tumor immunity, suggesting that NTRK3 may enhance immune surveillance and contribute to a more favorable immune landscape in BC [5]. Furthermore, the association between high NTRK3 expression with low CD4+ T cell infiltration and poorer prognosis emphasizes the importance of the tumor-immune interaction in BC progression. CD4+ T cells are pivotal for orchestrating adaptive immunity, and their low infiltration in the context of high NTRK3 expression may suggest an immunosuppressive microenvironment that favors tumor progression [16]. This finding underscores the complex role of NTRK3, where its high expression alone does not guarantee prognostic value, as it depends on the specific immune cell profile present.

The limitations of this study are as follows. First, our analysis relies on big data and does not include experimental validation or clinical trials. Second, although we observed a correlation between *NTRK3* expression and tumor progression, we lack detailed mechanistic insights into how NTRK3 influences BC. Conflicting findings regarding survival outcomes linked to *NTRK3* expression suggest a lack of consensus on its role in cancer progression, highlighting the need for further research.

## 5. Conclusions

Our findings demonstrate that *NTRK3* may be a potential prognostic biomarker in BC, highlighting its importance in both tumor progression and patient survival. To confirm this hypothesis, translational research using patient tissue samples, combined with in vitro cell experiments, will be essential. By examining NTRK3 expression in clinical BC samples and correlating it with clinical outcomes, we can assess its potential as a prognostic biomarker. Additionally, functional studies using BC cell lines, including NTRK3 knockdown or overexpression experiments, will help elucidate its mechanistic role in tumor growth, immune evasion, and metastasis. These combined approaches will provide a deeper understanding of NTRK3′s involvement in BC and support the development of targeted therapeutic strategies based on its expression and immune interaction profiles.

## Figures and Tables

**Table 1:** Clinical characteristics of NTRK3 mRNA expression in breast cancer.

	NTRK3		
	High	Low	*p*-Value
Age			
Gender			0.13
Male	3 (27.3)	8 (72.7)	
Female	50 (50.3)	498 (49.7)	
T stage			0.002
T1	144 (55.0)	118 (45.0)	
T2	275 (47.1)	309 (52.9)	
T3	72 (59.0)	50 (41.0)	
T4	10 (28.6)	25 (71.4)	
N stage			0.09
N0	248 (52.5)	224 (47.5)	
N1	167 (49.9)	168 (50.1)	
N2	45 (39.8)	68 (60.2)	
N3	38 (54.3)	32 (45.7)	
M stage			0.40
M0	413 (49.5)	422 (50.5)	
M1	8 (40.0)	12 (60.0)	
Stage			0.22
I	97 (56.7)	74 (43.3)	
II	279 (48.9)	292 (51.1)	
III	110 (48.7)	116 (51.3)	
IV	7 (38.9)	11 (61.1)	
Race			0.011
White	385 (55.4)	360 (44.6)	
Black	68 (40.0)	102 (60.0)	
Asian	23 (41.1)	33 (58.9)	
Pathology			<0.001
Invasive ductal carcinoma	200 (42.7)	268 (57.3)	
Invasive lobular carcinoma	99 (80.5)	24 (19.5)	
Mixed	38 (49.4)	39 (50.)	
Subtype			<0.001
Luminal A	303 (61.5)	190 (38.5)	
Luminal B	41 (22.9)	138 (77.1)	
HER2-enriched	10 (14.1)	61 (85.9)	
Triple negative	96 (57.1)	72 (42.9)	

## Data Availability

The data presented in this study are available on request from the corresponding author.

## Abbreviations

The following abbreviations are used in this manuscript:

NTRK	Neurotrophic receptor tyrosine kinase
BC	Breast cancer
TRK	Tropomyosin receptor kinase
TIICs	Tumor-infiltrating immune cells
TIMER	Tumor ImTmune Estimation Resource
OS	Overall survival
PFI	Progression-free interval
DSS	Disease-specific survival
TCGA	The Cancer Genome Atlas
HRs	Hazard ratios

## References

[1] Hamann U., Ankel C. (2018). Mammakarzinom: Diagnostik und Therapie—Das Wichtigste für den Internisten [Breast cancer: Diagnostics and therapy—The most important facts for internists]. Dtsch. Med. Wochenschr..

[2] World Health Organization (2024). Breast Cancer.

[3] Chetty R. (2019). Neurotrophic tropomyosin or tyrosine receptor kinase (NTRK) genes. J. Clin. Pathol..

[4] Drilon A., Laetsch T.W., Kummar S., DuBois S.G., Lassen U.N., Demetri G.D., Nathenson M., Doebele R.C., Farago A.F., Pappo A.S. (2018). Efficacy of larotrectinib in *TRK* fusion-positive cancers in adults and children. N. Engl. J. Med..

[5] Kue C.S., Kamkaew A., Voon S.H., Kiew L.V., Chung L.Y., Burgess K., Lee H.B. (2016). Tropomyosin receptor kinase C targeted delivery of a peptidomimetic ligand-photosensitizer conjugate induces antitumor immune responses following photodynamic therapy. Sci. Rep..

[6] Jin W., Kim G.M., Kim M.S., Lim M.H., Yun C., Jeong J., Nam J.-S., Kim S.-J. (2010). TrkC plays an essential role in breast tumor growth and metastasis. Carcinogenesis.

[7] Walker A. (2020). Neurotrophic tyrosine kinase inhibitors: A review of implications for patients, clinicians and healthcare services. J. Oncol. Pharm. Pract..

[8] Meng L., Yue X., Zhou D., Li H. (2020). Long non coding RNA OIP5AS1 promotes metastasis of breast cancer via miR3405p/ZEB2 axis. Oncol. Rep..

[9] Li T., Fu J., Zeng Z., Cohen D., Li J., Chen Q., Li B., Liu X.S. (2020). TIMER2.0 for analysis of tumor-infiltrating immune cells. Nucleic Acids Res..

[10] Kim H.R., Kim J., Woo H.J., Park M.S. (2024). TMEM14C is a novel biomarker for prognosis and diagnosis of liver hepatocellular carcinoma. Anat. Biol. Anthropol..

[11] Feng Y., Spezia M., Huang S., Yuan C., Zeng Z., Zhang L., Ji X., Liu W., Huang B., Luo W. (2018). Breast cancer development and progression: Risk factors, cancer stem cells, signaling pathways, genomics, and molecular pathogenesis. Genes. Dis..

[12] Stratton M.R., Campbell P.J., Futreal P.A. (2009). The cancer genome. Nature.

[13] Nik-Zainal S., Davies H., Staaf J., Ramakrishna M., Glodzik D., Zou X., Martincorena I., Alexandrov L.B., Martin S., Wedge D.C. (2016). Landscape of somatic mutations in 560 breast cancer whole-genome sequences. Nature.

[14] Beltjens F., Molly D., Bertaut A., Richard C., Desmoulins I., Loustalot C., Charon-Barra C., Courcet E., Bergeron A., Ladoire S. (2021). ER−/PR+ breast cancer: A distinct entity, which is morphologically and molecularly close to triple-negative breast cancer. Int. J. Cancer.

[15] Filippi A., Mocanu M.M. (2023). Mining TCGA database for genes with prognostic value in breast cancer. Int. J. Mol. Sci..

[16] He S., Ji Z., Zhang Q., Zhang X., Chen J., Hu J., Wang R., Ding Y. (2023). Investigation of *LGALS2* expression in the TCGA database reveals its clinical relevance in breast cancer immunotherapy and drug resistance. Sci. Rep..

[17] Luo Y., Kaz A.M., Kanngurn S., Welsch P., Morris S.M., Wang J., Lutterbaugh J.D., Markowitz S.D., Grady W.M. (2013). *NTRK3* is a potential tumor suppressor gene commonly inactivated by epigenetic mechanisms in colorectal cancer. PLoS Genet..

[18] Kamiya A., Inokuchi M., Otsuki S., Sugita H., Kato K., Uetake H., Sugihara K., Takagi Y., Kojima K. (2016). Prognostic value of tropomyosin-related kinases A, B, and C in gastric cancer. Clin. Transl. Oncol..

[19] Greco A., Miranda C., Pierotti M.A. (2010). Rearrangements of NTRK1 gene in papillary thyroid carcinoma. Mol. Cell Endocrinol..

[20] Vaishnavi A., Capelletti M., Le A.T., Kako S., Butaney M., Ercan D., Mahale S., Davies K.D., Aisner D.L., Pilling A.B. (2013). Oncogenic and drug-sensitive *NTRK1* rearrangements in lung cancer. Nat. Med..

[21] Wu G., Diaz A.K., Paugh B.S., Rankin S.L., Ju B., Li Y., Zhu X., Qu C., Chen X., Zhang J. (2014). The genomic landscape of diffuse intrinsic pontine glioma and pediatric non-brainstem high-grade glioma. Nat. Genet..

[22] Xu X., Tahan S.R., Pasha T.L., Zhang P.J. (2003). Expression of neurotrophin receptor Trk-C in nevi and melanomas. J. Cutan. Pathol..

[23] Bouzas-Rodriguez J., Cabrera J.R., Delloye-Bourgeois C., Ichim G., Delcros J.G., Raquin M.A., Rousseau R., Combaret V., Bénard J., Tauszig-Delamasure S. (2010). Neurotrophin-3 production promotes human neuroblastoma cell survival by inhibiting TrkC-induced apoptosis. J. Clin. Investig..

[24] Cosar R., Sut N., Ozen A., Tastekin E., Topaloglu S., Cicin I., Nurlu D., Ozler T., Demir S., Yıldız G. (2022). Breast cancer subtypes and prognosis: Answers to subgroup classification questions, identifying the worst subgroup in our single-center series. Breast Cancer Targets Ther..

[25] Kumar N., Gann P.H., McGregor S.M., Sethi A. (2023). Quantification of subtype purity in Luminal A breast cancer predicts clinical characteristics and survival. Breast Cancer Res. Treat..

[26] Park S., Koo J.S., Kim M.S., Park H.S., Lee J.S., Lee J.S., Kim S.I., Park B.W. (2012). Characteristics and outcomes according to molecular subtypes of breast cancer as classified by a panel of four biomarkers using immunohistochemistry. Breast.

